# A light-responsive engineered probiotic system to alleviate anxiety via gut-brain axis

**DOI:** 10.3389/fbioe.2026.1864003

**Published:** 2026-10-08

**Authors:** Yuling Chang, Zhaofang Hang, Yuhan Zhang, Yuhao Zhu, Xingang Li, Xuejun Wang, Di Zhang

**Affiliations:** 1 Jinan Microecological Biomedicine Shandong Laboratory and Shandong First Medical University, Jinan, China; 2 Shandong Key Laboratory of Brain Health and Function Remodeling, Institute of Brain and Brain‐Inspired Science, Shandong University, Jinan, China

**Keywords:** anxiety disorder, engineered probiotic, gamma-aminobutyric acid, gut-brain axis, optogenetics

## Abstract

Anxiety disorders are highly prevalent worldwide, yet effective treatment strategies remain limited. Overactive gut–brain communication has been linked to anxiety-like behaviors, and modulation of this signaling offers a promising approach for anxiety reduction. Leveraging the controllable release and biocompatibility of engineered bacteria, we developed a near-infrared (NIR)-responsive probiotic system that releases gamma-aminobutyric acid (GABA) to directly modulate gut vagal sensory input, thereby alleviating anxiety-like behavior. The system was constructed using *Escherichia coli* Nissle 1917 (EcN) carrying a thermosensitive GABA synthesis plasmid (EcN-Gadabc), coated with polydopamine (pDA), and further conjugated with a pepT1 targeting peptide for small-intestinal targeting. This design enabled precise NIR-triggered GABA release in the gut, improved bacterial survival under simulated gastrointestinal conditions, and enhanced intestinal retention following oral administration. In mice subjected to repeated restraint stress, treatment with the engineered bacteria under NIR irradiation alleviated anxiety-like behaviors, remodeled the gut microbiota, reduced the relative abundance of *Clostridium,* decreased serum IL-1β, IL-6 and TNF-α, increased IL-10, and reduced c-fos–positive neurons in the amygdala, nucleus tractus solitarius, and locus coeruleus. Transcriptomic analysis further indicated alterations in pathways associated with neuronal excitability, ion channel activity and calcium signaling. These findings support a non-invasive and remotely controllable strategy for gut–brain axis modulation using engineered probiotics and highlight its potential for precision bacteriotherapy in anxiety-related disorders.

## 1 Introduction

Anxiety disorders are characterized by excessive fear, persistent anxiety, and avoidance of perceived or potential threatening stimuli (Stratton, 2025). Globally, approximately 301 million individuals suffer from anxiety-related illnesses (Mahima and Pentela, 2025). Despite this growing burden, current clinical interventions for anxiety remain limited, with prolonged treatment courses, resource dependence, adverse side effects and variable efficacy (Cuijpers et al., 2008; Cipriani et al., 2018). Therefore, there is an urgent need to develop safe and effective anti-anxiety strategies that can be tailored to individual needs.

The gut–brain axis, which enables bidirectional crosstalk between the brain and the gastrointestinal tract via integrated neural, immune, endocrine, and metabolic signaling, has been increasingly recognized as a key player in the pathophysiology of anxiety disorders (Chen et al., 2022). Among these, the vagus nerve constitutes a direct anatomical connection between the gastrointestinal tract and brainstem nuclei such as the nucleus tractus solitarius, which subsequently projects to emotion-related regions including the amygdala and locus coeruleus (Petrut et al., 2025; Bonaz et al., 2018). Accumulating evidence indicates that gut microbiota-derived signals can influence mood and anxiety through multiple mechanisms, some of which involve vagal sensory pathways (Bercik et al., 2011; Liu et al., 2021; Siopi et al., 2023). The gut–brain axis is now recognized as a complex bidirectional communication network, the function of which is mediated not by a single dominant pathway but through the intricate interplay of neural, immune, and metabolic signaling (Mayer, 2011; Cryan et al., 2020).

Gamma-aminobutyric acid (GABA) is the principal inhibitory neurotransmitter in the central nervous system and plays a critical role in mood regulation. Dysfunction of the GABAergic system is frequently observed in patients with anxiety and depression (Luscher et al., 2011; Schür et al., 2016; Liwinski et al., 2023). The gastrointestinal (GI) tract contains substantial amounts of GABA (Krantis, 2000). Studies have shown that probiotics such as *Lactobacillus* and *Bifidobacterium* can produce GABA locally in the GI tract (Strandwitz et al., 2019), and this elevation has been associated with reduced anxiety-like behavior in mice (Ikegami et al., 2024). Given the limited permeability of GABA across the blood–brain barrier (Boonstra et al., 2015), these observations suggest that gut-derived GABA may influence brain function through non-humoral pathways. Notably, vagal afferent terminals express abundant GABA receptors (Egerod et al., 2018), and GABA also exerts immunomodulatory effects by suppressing pro-inflammatory cytokine production and promoting anti-inflammatory responses (Bhat et al., 2010; Prud’homme et al., 2015). These observations suggest that gut-derived GABA may act through multiple peripheral mechanisms, including modulation of immune function and neural signaling along the gut–brain axis.

Engineered probiotics have emerged as promising live biotherapeutic products for modulating gut vagal afferent input, enabling continuous production and localized delivery of therapeutic molecules at high concentrations (Sadhu et al., 2025). Previous studies have demonstrated the feasibility of this approach (Pan et al., 2022; Wang et al., 2025). However, oral delivery of engineered bacteria remains challenging due to exposure to gastric acid, bile salts, and digestive enzymes (Sahoo et al., 2025; Chen et al., 2025); these limitations can be mitigated through surface nanoengineering strategies that enhance bacterial viability and therapeutic efficacy. In particular, polydopamine (pDA) is an attractive biomimetic coating material because of its strong adhesiveness, biocompatibility and versatile chemical reactivity (Muttiah and Hanafiah, 2025). Under mildly alkaline conditions, pDA readily self-polymerizes on bacterial surfaces to form a stable nanoshell, thereby enhancing resistance to harsh gastrointestinal environments and improving intestinal retention (Chen et al., 2025). Current probiotic delivery systems mostly rely on non-targeted strategies, such as pH-sensitive polymers and spores (Luo et al., 2022.), which suffer from insufficient targeting ability and difficulty in achieving precise spatiotemporal control. As a functional platform, the catechol and amino groups of pDA enable further functionalization with targeting ligands, such as pepT1 targeting peptide, to improve site-specific intestinal localization (Yang et al., 2020). Moreover, pDA possesses photothermal properties. Under near-infrared (NIR) irradiation, it efficiently converts light into heat, enabling an externally controllable stimulus-responsive behavior. This property has been successfully applied to regulate gene expression and molecular release from engineered bacteria in the gut (Yang et al., 2020; Wang et al., 2025).

Here in this study, we developed a nanomaterial-assisted engineered probiotic system for NIR-responsive and precision-controlled GABA delivery in the gut (Figure 1). This system integrates three core modules: (1) a thermosensitive *E. coli* chassis engineered for GABA synthesis; (2) a pDA nanocoating that provides gastrointestinal protection and enables photothermal conversion; (3) a small-intestinal-targeting pepT1 peptide conjugated to the pDA layer to enhance mucosal adhesion. We evaluated the therapeutic potential of this system in a mouse model of repeated restraint stress-induced anxiety and explored its potential mechanisms involving peripheral inflammation, neural activation, gut microbiota remodeling, and transcriptomic changes in emotion-related brain regions. This study provides a non-invasive and remotely controllable strategy for gut–brain axis-targeted intervention in anxiety-related disorders.

**FIGURE 1:**
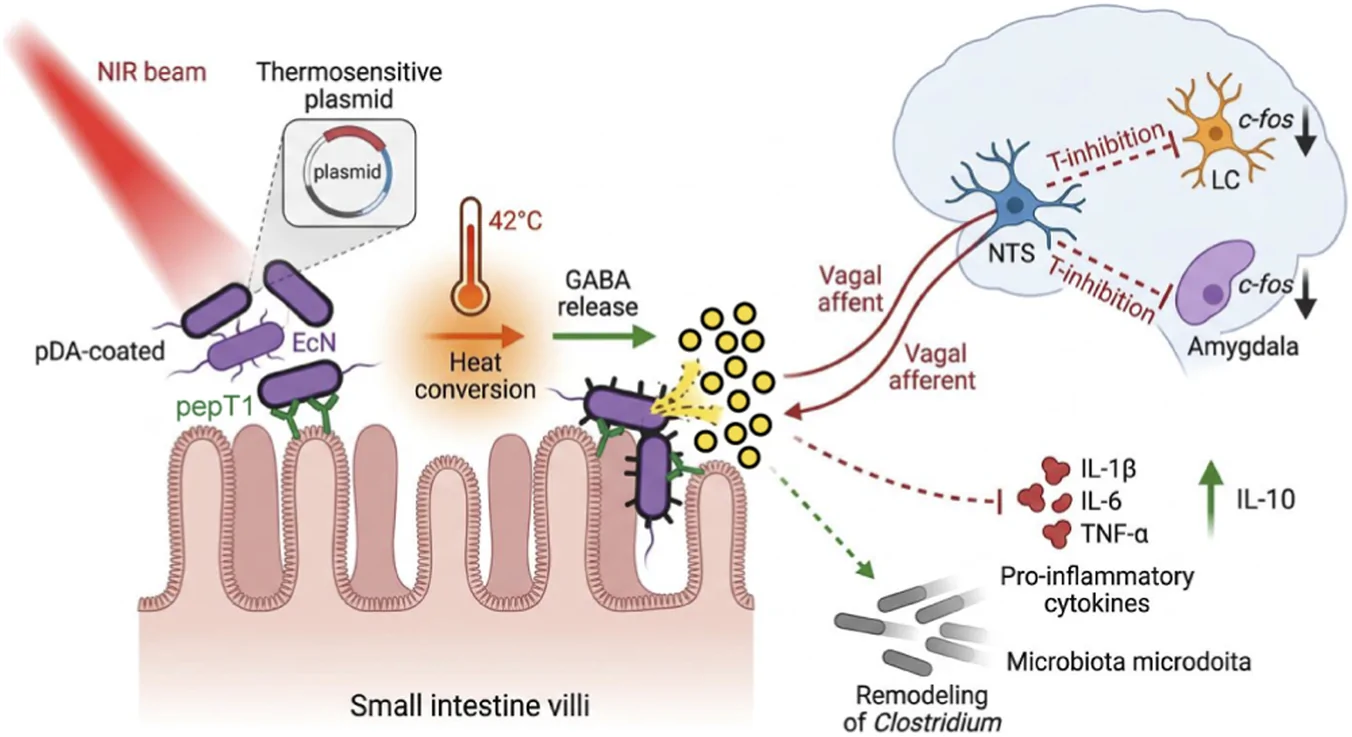
Schematic illustration of EcN-Gadabc@pDA-pepT1 mechanism for regulating anxiety-like behaviors via Gut-Brain Axis.

## 2 Materials and methods

### 2.1 Materials

The main chemicals and biological materials used in this study were as follows: LB Broth (LB, Hopebio), Kanamycin Solution (Solarbio), 0.01 M PBS (Solarbio), Agar (Solarbio), 10 mM Tris-HCl buffer (pH 8.5, sterile, Baiao Leibo), dopamine hydrochloride (Sigma-Aldrich), trypsin (Sigma-Aldrich), pepsin (Thermo Fisher Scientific), bile salts (Sigma-Aldrich), Cell Count Kit-8 (CCK-8, Beyotime), pepT1 targeting peptide (Santa Cruz Biotechnology). Simulated gastric fluid (SGF) and simulated intestinal fluid (SIF) were prepared as described in the United States Pharmacopeia. SGF was prepared by dissolving 2.0 g NaCl and 3.2 g pepsin in 1 L DI water and the pH was adjusted to 1.5 with HCl solution. SGF was filtered through a 0.22-µm membrane before use. SIF was prepared by dissolving 6.8 g KH_2_PO_4_ and 10 g trypsin in 1 L DI water and the pH was adjusted to 6.8 with NaOH solution. SIF was filtered through a 0.22-µm membrane before use.

### 2.2 Animals

Wild-type, specific pathogen-free (SPF) C57BL/6N male mice, 7 weeks old and weighing 24 ± 2 g, were purchased from Beijing Vital River Laboratory Animal Technology Co., Ltd. All mice were housed in an SPF animal facility under a cage-separation system, with no more than five mice per cage. The mice were fed an autoclaved rodent diet (Keao Xieli Feed Co., Ltd.) and given sterile water (autoclaved at 121 °C for 1.5 h). The housing environment was maintained at 20 °C–25 °C with a relative humidity of 30%–70%. After arrival, the mice were acclimatized under these conditions for 7 days before being used in experiments. Additionally, all the mice were immobilized in modified plastic syringes for 2 h each day for 14 days to induce repeated restraint stress. All experimental procedures were approved by the Animal Care and Use Committee of Jinan Microecological Biomedicine Provincial Laboratory.

### 2.3 Construction of plasmids and engineered bacteria

The engineered strain EcN-Gadabc, which carries the recombinant plasmid pBV220-Gadabc and secretes GABA, as well as the fluorescent reporter strain EcN-GFP, which carries the recombinant plasmid pBV220-GFP, were both constructed using *Escherichia coli* Nissle 1917 (EcN) as the chassis by the Jinan Microecological Biomedicine Provincial Laboratory (Wang et al., 2025).

### 2.4 Encapsulation of EcN with the poly-dopamine layer

10 mL of bacterial culture was collected and centrifuged. The supernatant was discarded, and the pellet was washed once with PBS by gentle pipetting. After differential centrifugation (8,000 rpm, 5 min), the supernatant was removed. The pellet was then resuspended in 10 mL of 10 mM Tris-HCl buffer containing 10 mg of dopamine hydrochloride (PH = 8.5). The mixture was mixed by pipetting and incubated in a shaking incubator (200 rpm, 37 °C) for 40 min. When the bacterial suspension turned grayish-white, the engineered bacteria EcN@pDA were obtained. Their morphology was observed using transmission electron microscopy. The size and zeta potential of EcN@pDA were measured by dynamic light scattering using a nanoparticle size and zeta potential analyzer.

### 2.5 External environment resistance assay for EcN@pDA

The protective effect of the pDA coating on EcN against simulated gastrointestinal conditions, including SGF, SIF, and bile salts (0.4%), was evaluated. For SGF resistance, equal amounts of EcN and EcN@pDA suspensions were separately added to SGF, mixed by pipetting and incubated in a shaking incubator (225 rpm, 37 °C). At 0, 0.5, 1 and 2 h, 50 µL samples were collected, washed with PBS, and then serially diluted 10-fold. The diluted samples were spread onto LB agar plates using a sterile spreader. The plates were incubated at 37 °C for 24 h, after which colonies were counted. For SIF and bile salt resistance, equal amounts of EcN and EcN@pDA suspensions were separately added to SIF and bile salt solution and incubated in a shaking incubator (225 rpm, 37 °C). At 0, 1, 2 and 4 h, 100 µL samples were collected, washed with PBS, and then resuspended in 100 µL PBS. Subsequently, 10 µL of CCK-8 solution was added, and the samples were incubated at 37 °C for 1 h. Cell viability was assessed by measuring the OD at 450 nm using a microplate reader.

### 2.6 Conjugation of the small-intestine-targeting peptide pepT1 to EcN@pDA

The EcN@pDA bacterial suspension was washed once with PBS by gentle pipetting, followed by differential centrifugation (8,000 rpm, 5 min). The supernatant was discarded and the pepT1 targeting peptide was added at a ratio of 10:1 (bacterial suspension to targeting peptide). The mixture was mixed by pipetting and incubated in a shaking incubator (200 rpm, 37 °C) for 30 min to obtain the engineered bacteria EcN@pDA-pepT1. Co-localization of the bacterial 488 green fluorescence and the targeting peptide 647 red fluorescence was observed using a laser scanning confocal microscope.

### 2.7 Evaluation of the intestinal adhesion of EcN@pDA-pepT1

The fluorescent reporter plasmid PAKgfpLux2, which allows imaging by a small animal *in vivo* imaging system (IVIS), was used to monitor the distribution of engineered bacteria in mice. The plasmid PAKgfpLux2 was introduced into EcN by electroporation and positive clones were selected using ampicillin (100 μg/mL). The resulting strain was named EcN-PAKgfpLux2 and used for subsequent experiments. Nine SPF wild-type C57BL/6N male mice (7 weeks old) were randomly divided into three groups (n = 3). Each mouse received 100 μL of one of the following engineered strains (1 × 10^9^ CFU) by oral gavage: EcN-PAKgfpLux2, EcN-PAKgfpLux2@pDA and EcN-PAKgfpLux2@pDA-pepT1. Bioluminescence intensity in the mice was measured using IVIS at 0, 4 and 8 h after administration. For the *ex vivo* intestinal adhesion assay, freshly dissected mouse small intestines were placed on Petri dishes, cut open longitudinally, and rinsed with a small amount of PBS. A defined amount of each engineered strain (EcN-PAKgfpLux2, EcN-PAKgfpLux2@pDA, EcN-PAKgfpLux2@pDA-pepT1; 1 × 10^9^ CFU) was added to the opened intestinal segments. After 1 h of incubation, the intestines were washed three times with PBS and bioluminescence intensity was measured using IVIS.

### 2.8 Near-infrared irradiation

To measure the photothermal effect of EcN@pDA, the solution was placed at the center of near-infrared (NIR) light irradiation (808 nm, 2 W/cm^2^). Temperature changes were recorded every 3 min over a total irradiation period of 15 min. Temperature was measured using a fiber-optic thermometer placed in direct contact with the bacterial suspension for *in vitro* measurements. Thermal images was recorded using a visual thermal imager (HIKMICRO, TPE09Pro). This device has an spatial resolution of 256 × 192 pixels and a thermal sensitivity of 0.05 °C.

Every 3 min, the temperature of the bacterial suspension and changes in thermal images were recorded using a fiber optic thermometer and a visual thermal imager.

### 2.9 Functional validation of EcN@pDA-pepT1 *in vivo*

Thirty-six wild-type SPF C57BL/6N male mice (7 weeks old) were used. After acclimation, mice were randomly assigned to three groups (n = 12 each), a control group (CTL, oral saline), a control-strain group (EcN-GFP, 1 × 10^9^ CFU per mouse) and an engineered-strain group (EcN-GABA, EcN-Gadabc@pDA-pepT1, 1 × 10^9^ CFU per mouse). All groups received daily oral gavage for 14 consecutive days, with free access to food and water. Following the gavage regimen, all mice underwent daily repeated restraint stress for 14 days to induce anxiety. For each restraint session, a mouse was placed in a modified 50 mL centrifuge tube (30 mm × 115 mm, with ventilation holes) for 2 h without food or water. After release, the mouse rested in its home cage for 2 h, then received a 40 min NIR light exposure on the abdomen (note: NIR irradiation was applied to all three groups, including CTL and EcN-GFP, under identical conditions), followed by another 40 min rest. On days 10, 12 and 14 of the anxiety induction period, after the daily procedures were completed, mice were tested in the following order (from lower to higher stress), open field test, elevated plus maze test and light-dark box test, to evaluate anxiety-like behaviors.

#### 2.9.1 Open field test (OFT)

The experimental apparatus was an open square box made of transparent acrylic (60 × 60 × 50 cm). The floor was divided into 9 equal squares, with the center square defined as the central area. At the beginning of the test, a mouse was placed in the central area and allowed to explore freely for 10 min. Recordings included the total distance traveled over the 10 min to assess locomotor activity, as well as the time spent in the central area and the number of entries into the central area, which reflect anxiety levels induced by the open space.

#### 2.9.2 Elevated plus maze test (EPM)

The apparatus consisted of two open arms (30 × 5 cm) and two closed arms (30 × 5 × 15 cm), with a central platform (5 × 5 cm) at the junction. The entire setup was elevated 70 cm above the floor. At the beginning of the test, a mouse was placed on the central platform facing the corner between an open arm and a closed arm and allowed to explore freely for 5 min. Recordings included the time spent in the open arms, the number of entries into the open arms and the total number of arm entries, which were used to assess anxiety levels induced by the elevated, open environment.

#### 2.9.3 Light-dark box (LDB)

The apparatus consisted of a light compartment and a dark compartment. The dark compartment occupied one-third of the total volume, and the light compartment occupied two-thirds. A small opening (5 × 7 cm) was made in the lower part of the divider between the two compartments, allowing the mouse to move freely between them. At the start of the test, a mouse was placed in the dark compartment facing the wall, the lid was closed and the mouse was allowed to explore freely for 5 min. Recordings included the latency to first enter the light compartment, the total number of entries into the light compartment and the total time spent in the light compartment, which were used to assess anxiety levels induced by the brightly lit environment.

### 2.10 Amygdala transcriptomic analysis

After behavioral testing, amygdala tissues were collected from mice in the CTL (oral saline, n = 6), the GFP (n = 7) and the GABA (n = 6) groups. Total RNA was extracted using TRIzol and RNA quality was assessed by agarose gel electrophoresis and a NanoDrop spectrophotometer. Samples meeting the criteria of OD 260/280 = 1.8–2.2, concentration ≥ 400 ng/μL and RIN ≥ 7.0 were used for library construction. mRNA was enriched, fragmented, reverse-transcribed into cDNA, subjected to end repair, A-tailing, adaptor ligation and PCR amplification. After quality control with an Agilent 2,100 Bioanalyzer, qualified libraries were used for paired-end sequencing by Majorbio Bio-Pharm Technology Co., Ltd. Raw data were processed for quality control and aligned to the reference genome and gene expression was quantified using the Majorbio cloud platform. DEGs were identified with |log2FoldChange| ≥ 1 and adjusted *P* < 0.05. Volcano plots, Venn diagrams and GO enrichment analyses were used to characterize differential expression patterns and potential biological functions.

### 2.11 Gut microbiota analysis

To investigate the effect of the engineered bacterial intervention on the gut microbiota composition of mice, fresh fecal samples were collected from each group on day 14 of the oral gavage treatment. The samples were immediately snap-frozen in liquid nitrogen and then transferred to a −80 °C freezer for storage until further processing. 16S rRNA sequencing analysis of the fecal samples was performed by Hangzhou Lianchuan Biotechnology Co., Ltd. Subsequent bioinformatics analysis was carried out on the Lianchuan Microbiome Cloud Analysis Platform.

### 2.12 Statistical analysis

All experimental data were processed and graphs were generated using GraphPad Prism 10.1.2. Quantitative data are presented as mean ± standard error of the mean (SEM). For comparisons among multiple groups, one-way ANOVA was used. When homogeneity of variance was assumed, *post hoc* pairwise comparisons were performed using the least significant difference (LSD) t-test. Comparisons between two groups were made using the unpaired t-test. A P value <0.05 was considered statistically significant. Behavioral data from the open field test, elevated plus maze test and light-dark box test were automatically collected and exported using the Anymaze behavioral analysis system. To ensure transparency in statistical reporting, we have compiled comprehensive statistical tables for all endpoints, including behavioral tests (OFT, EPM, LDB), serum cytokine levels (IL-1β, IL-6, TNF-α, IL-10), and c-fos immunohistochemistry. For each comparison, we report exact P-values, F-statistics, and *post hoc* pairwise results (CTL vs. EcN-GFP, CTL vs. EcN-GABA, and EcN-GFP vs. EcN-GABA). These tables have been added as Supplementary Table S1, allowing readers to fully evaluate the statistical basis for our conclusions.

## 3 Results

### 3.1 Assembly of photothermal nanomaterials onto EcN

To achieve precise spatiotemporal control over GABA release, we modified the thermosensitive EcN-Gadabc strain with the photothermal nanomaterial pDA on its surface. At pH 8.5, dopamine underwent oxidative self-polymerization to form pDA nanoparticles. The amino and catechol groups on the pDA surface formed covalent or non-covalent interactions with the bacterial cell wall components, thereby anchoring pDA onto the bacterial surface to generate the EcN-Gadabc@pDA (Figure 2A). Transmission electron microscopy images confirmed that pDA was successfully anchored onto the probiotic surface (Figure 2B). Compared with unmodified probiotics, the average particle size of the complex increased from 1,007 nm to 1,429 nm and the average surface zeta potential shifted from −18.9 mV to −13.1 mV (Figure 2C). The increase in particle size was attributed to the pDA coating, while the positive shift in surface potential was associated with the amino groups present in pDA. Under NIR light irradiation, the temperature of the suspension of EcN@pDA significantly increased by 3 °C–8 °C (Figures 2D,E), exhibiting the efficacy of the photothermal effect. The temperature changes of the EcN@pDA suspension remained stable throughout three cycles of NIR irradiation and natural cooling (Figure 2F), exhibiting the stability of the photothermal effect. The photothermal effect of EcN@pDA is strictly dependent on NIR irradiation. When NIR light is turned off, the temperature returns to baseline immediately due to the absence of continuous energy input and the efficient heat dissipation in the aqueous environment (Supplementary Figure S1) and gene induction can be achieved at 42 °C (Supplementary Figure S2). Next, using the green-fluorescent strain EcN-GFP@pDA, we observed that NIR irradiation significantly enhanced bacterial fluorescence intensity after 30 and 60 min (Figures 2G,H). Meanwhile, the concentration of GABA produced by EcN-Gadabc@pDA was significantly higher after 60 min of NIR irradiation than that in the non-irradiated control group (Figure 2I). Together, these results demonstrate the successful construction of a photothermally responsive engineered strain, EcN-Gadabc@pDA, in which the pDA nanoparticles on the probiotic surface can stably receive optical signals and convert them into thermal signals. Then, we measured serum GABA concentrations in mice following 14 days of oral administration of EcN-Gadabc@pDA-pepT1 with or without NIR irradiation. As shown in the newly added Supplementary Figure S3, serum GABA levels were significantly elevated in the NIR-irradiated group compared to the non-irradiated control. These data provide direct *in vivo* evidence that the NIR-responsive system effectively triggers GABA release in the gut, which subsequently enters the circulation.

**FIGURE 2:**
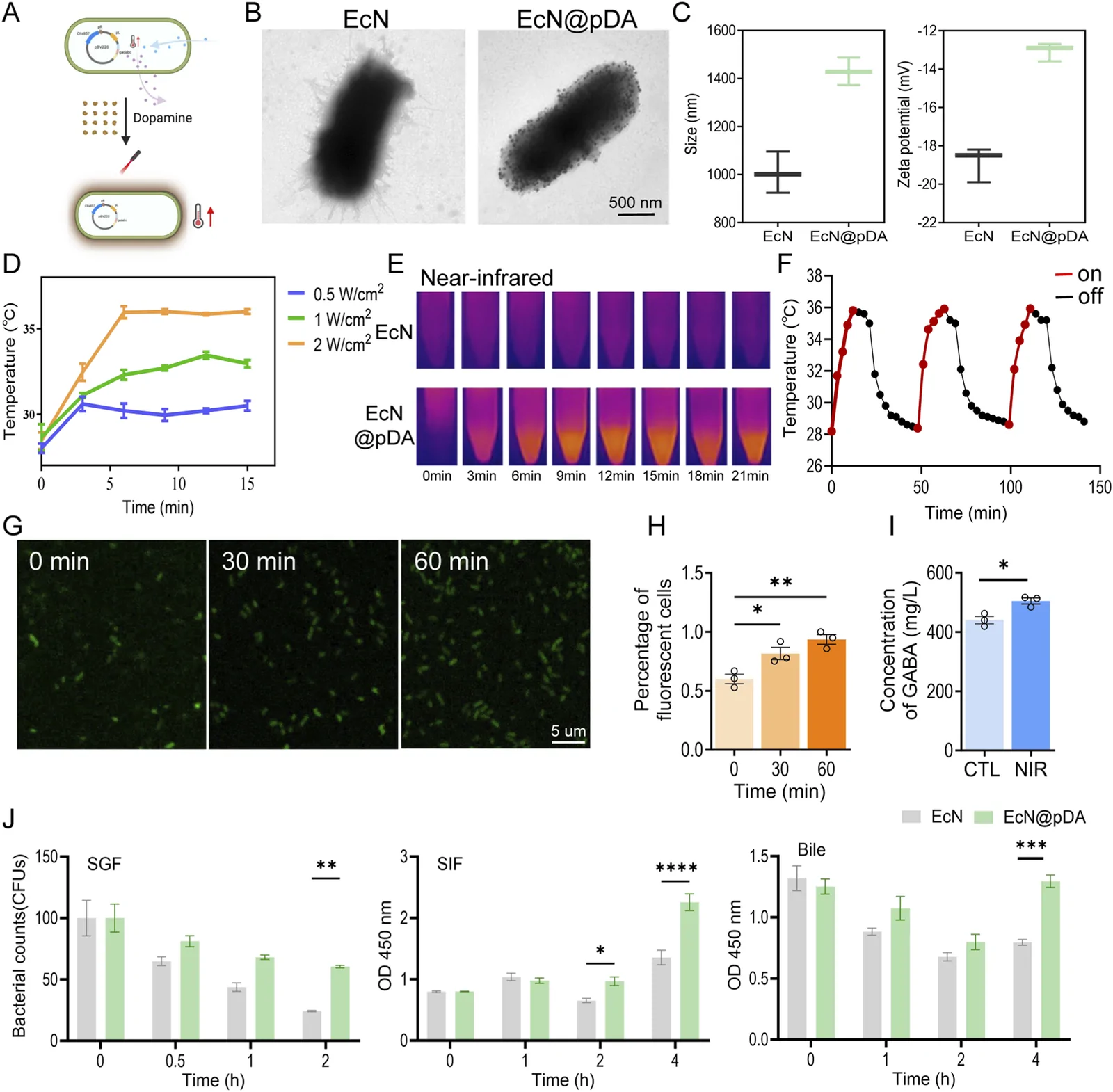
Assembly of pDA onto EcN. **(A)** Schematic illustration of pDA coating on the bacterial surface. **(B)** TEM images of EcN and EcN@pDA, scale bar: 0.5 μm. **(C)** Size and zeta potential of EcN and EcN@pDA. **(D)** Temperature changes of EcN@pDA suspensions under near-infrared (NIR) irradiation at different power levels. **(E)** Infrared thermal images of EcN and EcN@pDA suspensions upon NIR stimulation. **(F)** Temperature changes of EcN@pDA suspensions over three cycles of NIR stimulation followed by natural cooling. **(G)** Fluorescence images of EcN‐GFP@pDA after 0, 30, and 60 min of NIR stimulation, scale bar: 5 μm. **(H)** Percentage of fluorescent cells after 0, 30 and 60 min of NIR treatment. **(I)** GABA concentrations secreted by EcN-Gadabc@pDA with or without NIR stimulation. **(J)** Survival rates of EcN and EcN@pDA in SGF, SIF and bile. All data are presented as mean ± SEM. *P < 0.05, **P < 0.01.

The strongly acidic gastric environment in the gastrointestinal tract easily inactivates most probiotics. Subsequent degradation induced by bile salts and digestive enzymes further compromises bacterial survival. The assembly of the pDA layer did not adversely affect the normal growth or viability of the probiotics (Supplementary Figure S4), indicating the pDA coating had a minimal effect on the growth of EcN. We next tested whether the pDA coating could protect the probiotics under gastrointestinal conditions. After 2 h of SGF treatment, the survival rate of EcN@pDA was significantly higher than that of uncoated EcN-Gadabc. Similarly, under SIF and bile salt treatment, EcN@pDA showed higher survival rates after 4 h (Figure 3J). Together, these results confirm that the pDA coating can serve as an effective protective layer for oral delivery, providing technical support for engineered bacteria to reach the gut via the oral route and exert their functions.

**FIGURE 3:**
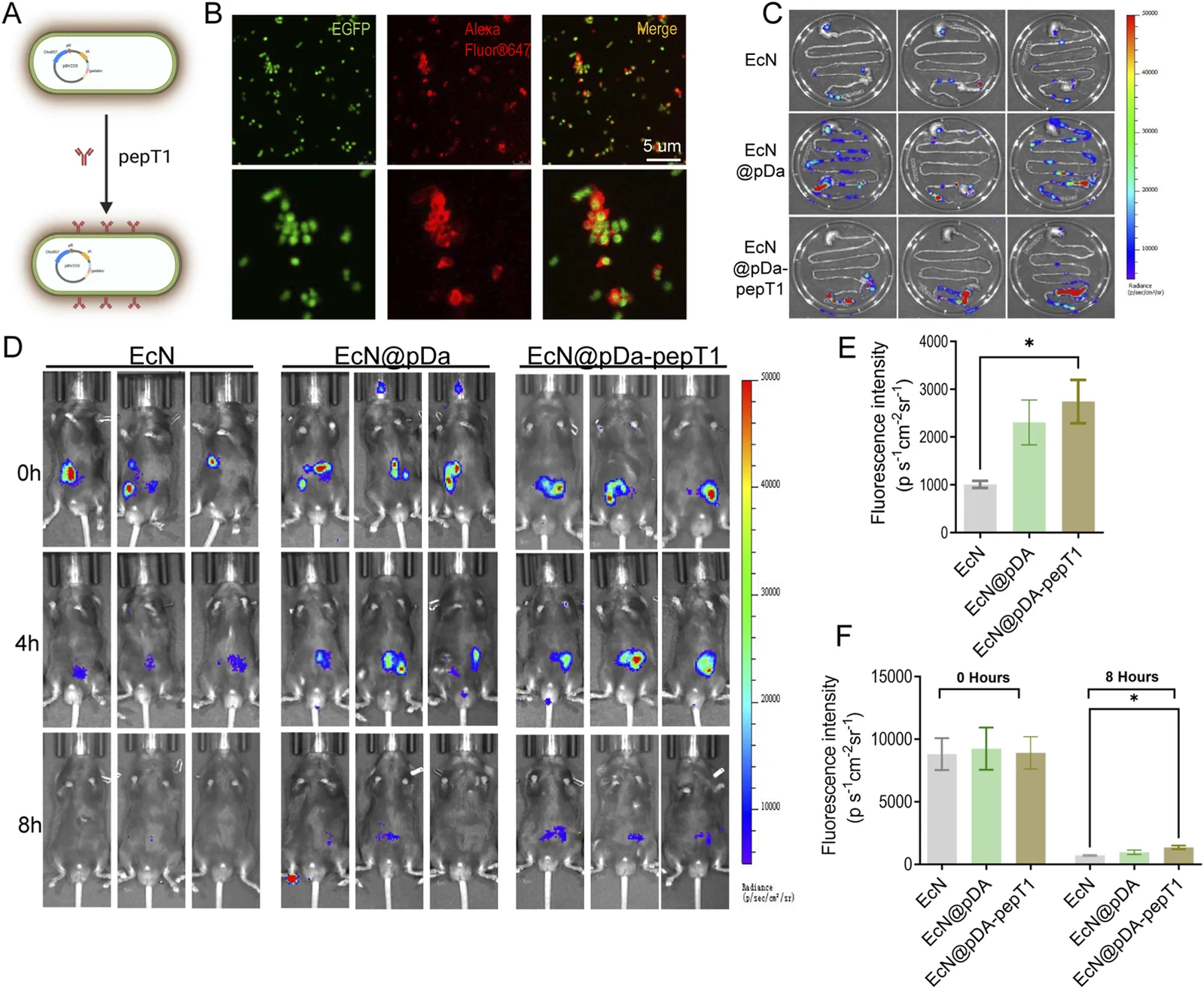
Assembly of pepT1 onto EcN@pDA. **(A)** Schematic illustration of pepT1 targeting peptide conjugation onto the EcN@pDA surface. **(B)** Co-localization images of Alexa Fluor®647 pepT1 targeting peptide and EcN-GFP@pDA. **(C)** Bioluminescence images of *ex vivo* intestines after incubation with EcN, EcN@pDA and EcN@pDA-pepT1. **(D)** Bioluminescence images of mice at different time points following gavage with EcN, EcN@pDA and EcN@pDA-pepT1. **(E)** Analysis of fluorescence intensities of the intestines. **(F)** Bioluminescence intensity at 0 and 8 h in mice gavaged with EcN, EcN@pDA, and EcN@pDA-PepT1. All data are presented as mean ± SEM. *P < 0.05, **P < 0.01, ***P < 0.001, ****P < 0.0001.

### 3.2 Assembly of the intestine-targeting peptide onto EcN@pDA

To enhance intestinal adhesion and retention of the engineered bacteria, we conjugated the small-intestine-targeting pepT1 peptide onto the surface of EcN@pDA to generate EcN@pDA-pepT1 (Figure 3A). As shown in Figure 3B, the red fluorescence signals from pepT1 targeting peptide (647 nm channel) surrounded the green fluorescence derived from EcN@pDA (488 nm channel), with obvious colocalization, which confirmed the successful conjugation of pepT1 targeting peptide onto the pDA-modified bacterial surface. Subsequently, EcN, EcN@pDA and EcN@pDA-pepT1 were co-incubated with freshly isolated mouse intestinal tissues in phosphate-buffered saline (PBS) for 1 h. After thorough washing with PBS three times, intestinal fluorescent signals were detected using an IVIS imaging system. Quantitative fluorescence analysis revealed that the fluorescence of EcN@pDA-pepT1 was highly enriched in the terminal small intestine, demonstrating that pepT1 modification effectively endows engineered EcN with targeted intestinal colonization capacity (Figures 3C,E). To monitor the distribution and retention of the engineered bacteria *in vivo*, mice were separately gavaged with EcN, EcN@pDA and EcN@pDA-pepT1. Eight hours after oral gavage, the EcN@pDA-pepT1 group maintained distinctly higher bioluminescence intensity compared with the EcN and EcN@pDA groups (Figures 3D,F). Immunofluorescence imaging further revealed direct attachment of pepT1-decorated bacteria to the small intestinal villi (Supplementary Figure S5). These results verified that pepT1 modification could strengthen the intestinal mucosal adhesion of engineered EcN and effectively retard bacterial clearance in the intestinal tract.

### 3.3 Alleviation of anxiety-like behaviors by EcN@pDA-pepT1 in mice

To explore the regulatory effects of the engineered strain EcN-Gadabc@pDA-pepT1 on anxiety-like behaviors *in vivo*, mice were randomly assigned to three experimental groups. Animals received daily intragastric administration for 14 days with an equal volume of sterile saline (CTL group), the control strain EcN-GFP@pDA-pepT1 (EcN-GFP group), or the engineered strain EcN-Gadabc@pDA-pepT1 (EcN-GABA group) (Figure 4A). In the EPM test, compared with the EcN-GFP group, the EcN-GABA group showed a significant increase in time spent in the open arms (Figure 4B). Moreover, the total number of arm entries were also significantly increased, indicating that the engineered probiotic systemEcN-Gadabc@pDA-pepT1 alleviated the height-induced anxiety. In the OFT test, the EcN-GABA group showed a significant increase in the number of entries into the center zone and the total distance traveled (Figure 4C), along with increasing trends in time spent in the center zone, further confirming the anxiolytic effects of the engineered probiotic EcN-Gadabc@pDA-pepT1 in an open space. In the LDB test, the EcN-GABA group showed a significant increase in the number of entries into the light box (Figure 4D), together with a decreasing trend in the latency to first entry. We have calculated Cohen’s d effect sizes for all primary behavioral endpoints, the effect sizes for all behavioral parameters range from 0.95 to 1.32, all falling within the “arge” effect size category (Cohen’s d ≥ 0.8) according to Cohen’s criteria (Supplementary Table S2). These results indicated that the engineered probiotic system EcN-Gadabc@pDA-pepT1, together with NIR stimulation, exhibits an overall anti-anxiety trend across multiple behavioral assays.

**FIGURE 4:**
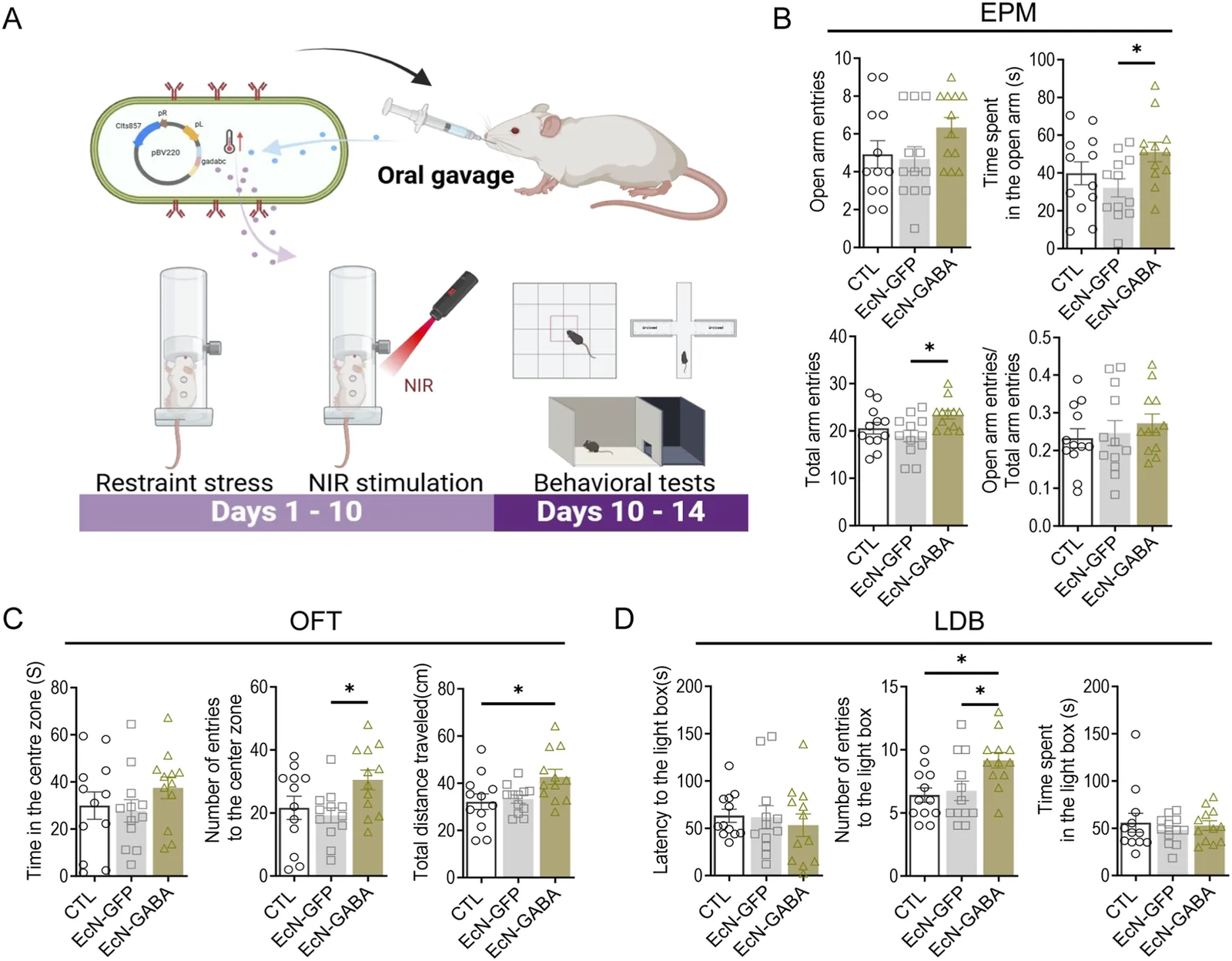
*In vivo* functional assessment of engineered EcN-Gadabc@pDA-pepT1 in mice. **(A)** Schematic illustration of the experimental setup. **(B)** Open arm exploration frequency, time spent in open arms, percentage of open arm entries, and total entries into open and closed arms. **(C)** Time spent in the center zone, number of center entries, and total distance traveled in the open field. **(D)** Latency to enter the light box, number of light-box explorations and time spent in the light box. All data are shown as mean ± SEM. *P < 0.05.

To preliminarily assess the biosafety of the engineered probiotic system, we performed serum biochemical analyses on mice after 14 days of daily oral administration of EcN-Gadabc@pDA-pepT1 plus NIR irradiation. As shown in Supplementary Figure S6, serum levels of alanine aminotransferase (ALT), albumin (ALB), blood urea nitrogen (BUN), Creatinine (CREA), and Creatine Kinase (CK), were all within the normal range, indicating no overt acute hepatic, renal or myocardial damage following the applied treatment regimen.

### 3.4 Modulation of gut microbiota composition by EcN@pDA-pepT1

Considering that probiotics confer multiple beneficial effects on the host through gut microbiota modulation and accumulating evidence has demonstrated a strong correlation between gut microbes and anxiety disorders, we further systematically analyzed the diversity, community structure and composition of intestinal microbiota. The Pielou evenness, Simpson and Shannon indices were calculated to evaluate the effects of the engineered strain EcN-Gadabc@pDA-pepT1 on the alpha diversity of gut microbiota. The results revealed that combined treatment with EcN-Gadabc@pDA-pepT1 and NIR irradiation partially rescued the decline in gut microbial diversity induced by EcN-GFP administration coupled with NIR exposure (Figure 5A). Furthermore, principal coordinate analysis (PCoA) and principal component analysis (PCA) were performed to reduce the dimensionality of the microbial community structure data and to visualize the differences among groups. PCoA showed that the first two axes explained 37.72% of the variance among samples (Figure 5B), while PCA showed that the first two axes explained 79.56% of the variance. We next analyzed the relative abundance of gut microbiota at the genus level. The relative abundance profiles of the top 30 genera differed across the three groups (Figure 5C). Compared with the CTL group, the EcN-GABA treatment group showed a significant reduction in the relative abundance of *Clostridium* in the gut (Figure 5D). We performed PICRUSt2 functional prediction analysis on the 16S rRNA sequencing data. As shown in Supplementary Figure S7, the expression levels of Alpha-mannosidase and Polygalacturonase were significantly upregulated. Overall, the engineered strain, as an exogenous probiotic, reshaped the gut microbiota.

**FIGURE 5:**
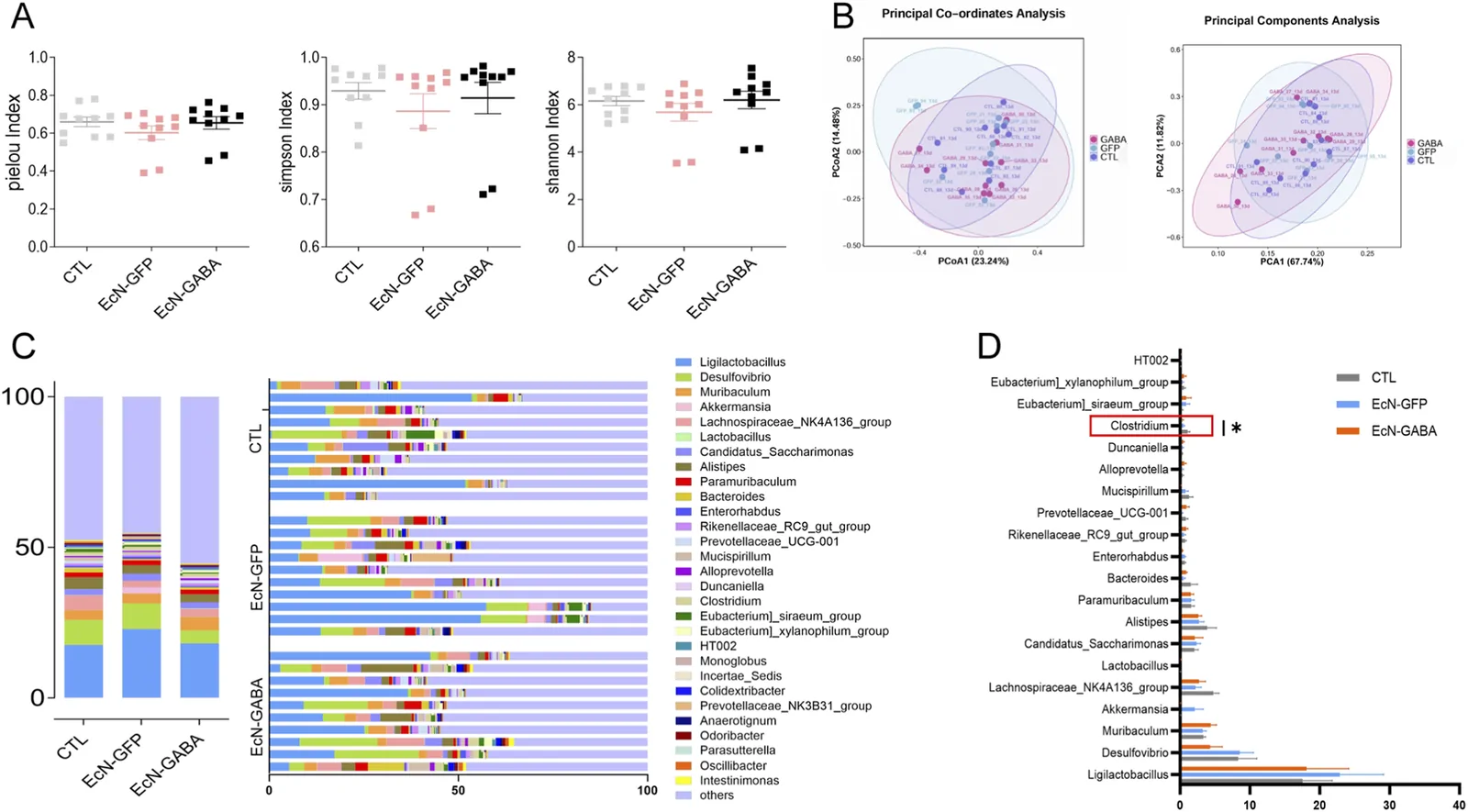
Changes in gut microbiota following EcN-Gadabc@pDA-pepT1/NIR treatment. **(A)** Alpha diversity of the gut microbiota. **(B)** Beta diversity of the gut microbiota. **(C)** Top 30 taxa at the genus level in composition analysis. **(D)** Top 20 genera with significant differences. All data are shown as mean ± SEM. *P < 0.05.

### 3.5 Regulation of systemic inflammation and amygdala neuronal activity by EcN@pDA-pepT1

A well-defined correlation has been confirmed between anxiety-like behaviors and inflammatory status. Therefore, we further explored the effects of this engineered strain on serum inflammatory cytokine levels in mice. Compared with the CTL and EcN-GFP groups, the EcN-GABA treatment group had significantly lower serum levels of the pro-inflammatory cytokines IL-1β, IL-6, TNF-α and significantly higher levels of the anti-inflammatory cytokine IL-10 (Figure 6A). These findings suggested that the anti-anxiety effect of the EcN@pDA pepT1 may be closely related to its ability to rebalance inflammatory cytokines and alleviate neuroinflammation. To explore the neurobiological mechanisms underlying the anxiety-like behavior improvement mediated by EcN-Gadabc@pDA-pepT1, we used c-fos immunofluorescence staining to detect changes in neuronal activity in brain regions closely associated with anxiety regulation. As shown in Figure 6B, the EcN-GABA treatment group exhibited particularly pronounced changes in the amygdala, a core brain region for emotional processing. The numbers of c-fos-positive neurons in the basolateral amygdala (BLA), central amygdala (CeA) and medial amygdala (MeA) were significantly lower in the EcN-GABA group than in the CTL group, indicating that the EcN-Gadabc@pDA-pepT1 significantly suppressed neuronal activity in the amygdala and related emotional circuit brain regions, thereby exerting an anti-anxiety effect. In addition, we observed general activity changes in several other brain regions involved in mood and anxiety regulation, including the infralimbic cortex (IL), claustrum (Cl) and bed nucleus of the stria terminalis (BST) (Supplementary Figure S8), which exhibited a consistent tendency. To further corroborate the reduction of neuronal activity at the protein level, we performed ARC immunofluorescence staining, another well-established immediate early gene marker of neuronal activation. Consistent with the c-fos results, the number of ARC-positive neurons in the amygdala was also markedly decreased in the EcN-GABA treatment group compared with controls (Supplementary Figure S9). Collectively, these findings support that EcN Gadabc@pDA-pepT1 improves anxiety-like behaviors by regulating inflammation and attenuating activation of the amygdala and related mood-regulating brain regions.

**FIGURE 6:**
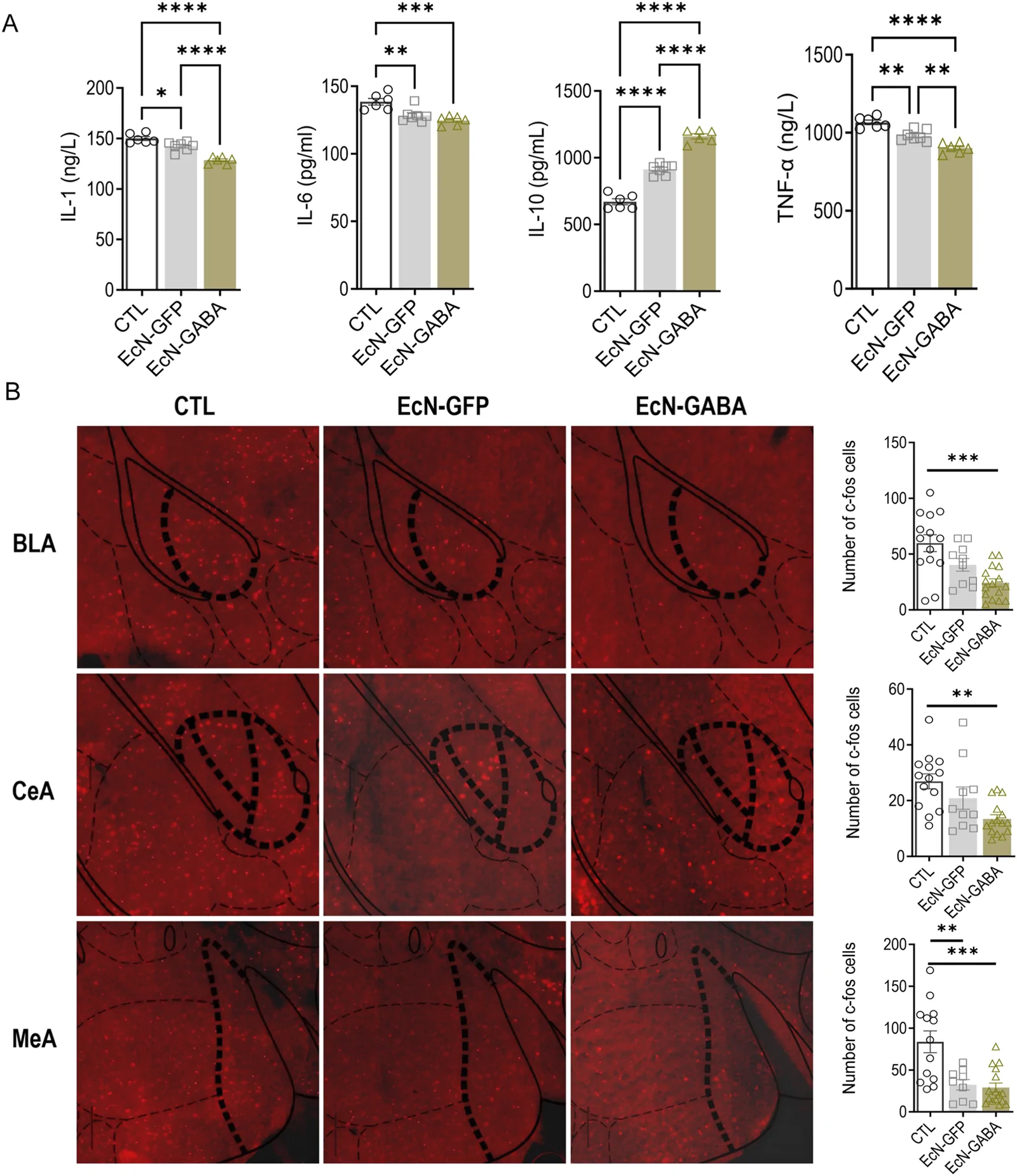
Changes in serum inflammatory factors and anxiety-related brain regions after EcN-Gadabc@pDA-pepT1/NIR treatment. **(A)** Levels of serum inflammatory factors in the CTL, EcN-GFP and EcN-GABA groups. **(B)** C-Fos positive cell counts in the amygdala of mice from the CTL, EcN-GFP and EcN-GABA groups. All data are presented as mean ± SEM. *P < 0.05, **P < 0.01, ***P < 0.001, ****P < 0.0001.

### 3.6 Transcriptomic alterations in the amygdala

Given the central role of the amygdala in emotional regulation and our prior results have verified that EcN Gadabc@pDA-pepT1 markedly suppresses local neuronal activity, we next targeted the amygdala to further explore the regulatory effects of this probiotic system on amygdala transcriptome profiles. Volcano plot analysis showed that in the GFP vs. CTL comparison, a total of 242 differentially expressed genes were identified, including 140 upregulated and 102 downregulated genes. In the GABA vs. GFP comparison, a total of 221 differentially expressed genes were identified, including 80 upregulated and 141 downregulated genes (Figure 7A). These results indicated that both colonization by the EcN-GFP and the release of GABA significantly affect amygdala gene expression. Notably, in the GABA group, most differentially expressed genes were downregulated. The significant downregulation of the potassium channel-related gene *Kcne2* indicated that GABA release may modulate neuronal excitability via the regulation of ion channels. Venn diagram analysis showed that among the upregulated genes, 129 were unique to the GFP vs. CTL comparison and 65 were unique to the GABA vs. GFP comparison, with no overlap between the two groups. Among the downregulated genes, 88 were unique to the GFP vs. CTL comparison and 130 were unique to the GABA vs. GFP comparison, again with no overlap (Figure 7B). These results revealed that the gene sets modulated by colonization of the control GFP strain and those altered by GABA release are completely distinct, suggesting that the two interventions function through divergent molecular mechanisms. GO enrichment analysis revealed marked differences in biological functions enriched by differentially expressed genes between the two groups (Figure 7C). In the GFP vs. CTL comparison, upregulated genes were enriched in biological processes associated with neuron differentiation, dopaminergic neuron differentiation, and the regulation of axon guidance, all of which participated in neuronal development. Downregulated genes in this comparison were enriched in functional terms, including neuropeptide activity and hormone activity. These findings indicated that colonization by the EcN strain alone indirectly modulated central nervous system function by regulating neuropeptide signaling and neuronal plasticity. In the GABA vs. GFP comparison, upregulated genes were enriched in processes such as the positive regulation of cytosolic calcium ion concentration and sensory neuron axon guidance. Downregulated genes were enriched in ion channel-related functions, encompassing transmembrane transporter activity and sodium ion transmembrane transporter activity. Notably, the significant downregulation of the potassium channel-related gene *Kcne2* corroborated the alterations in ion transport functions identified via GO enrichment analysis. This evidence further suggested that GABA release altered neuronal excitability through the modulation of ion channels.

**FIGURE 7:**
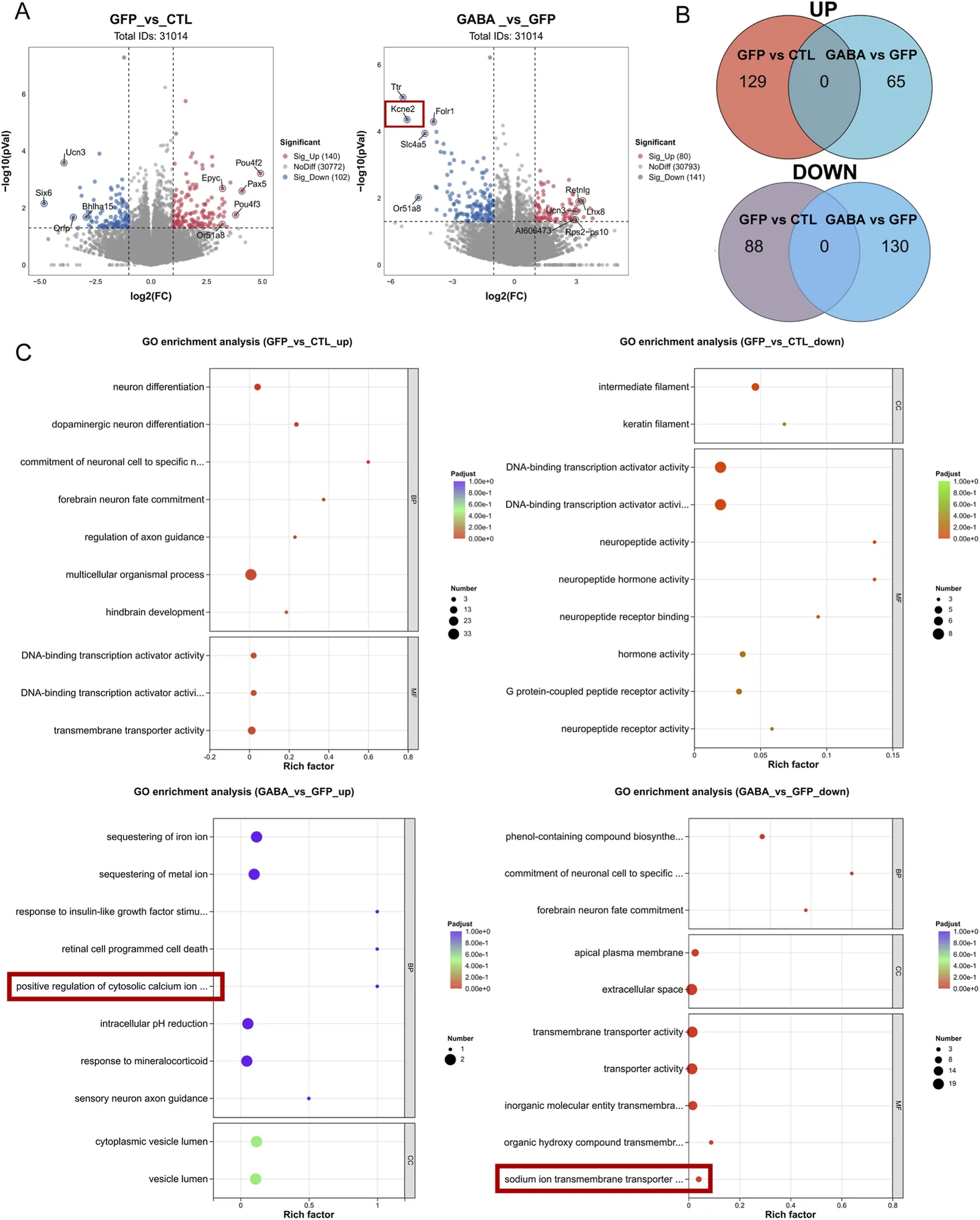
Amygdala transcriptome changes after EcN-Gadabc@pDA-pepT1/NIR treatment. **(A)** Volcano plots comparing GFP vs. CTL and GABA vs. GFP. **(B)** Venn diagram of the GFP vs. CTL and GABA vs. GFP comparisons. **(C)** GO enrichment analysis for the GFP vs. CTL and GABA vs. GFP groups. All data are presented as mean ± SEM. *P < 0.05.

## 4 Discussion

Probiotics, which naturally reside in the gastrointestinal tract, provide a practical platform for modulating gut–brain axis signaling. Here, we developed a nanomaterial-assisted, photothermally responsive engineered probiotic system, EcN-Gadabc@pDA-pepT1, which integrates thermosensitive gene regulation, pDA-mediated photothermal conversion and pepT1 targeting peptide-guided intestinal targeting. This system enabled precise and non-invasive spatiotemporal regulation of GABA delivery in the gastrointestinal tract and alleviated anxiety-like behaviors in a murine model of repeated restraint stress. Our findings suggest that this multimodal strategy enhances bacterial resilience against gastrointestinal barriers and exerts anxiolytic-like effects in association with coordinated changes in the gut microbiota, peripheral immune responses and central neural circuits. Owing to the favorable tissue penetration and non-invasive controllability of NIR light (Sun et al., 2024), this strategy avoids fiber-optic implantation and offers a promising approach for remote modulation of the gut–brain axis.

To achieve temperature-controlled GABA production, the gada-gadb-gadc cassette was introduced into EcN using the thermosensitive CIts857/pRpL-regulated pBV220 system (Wang et al., 2025). Consistent with our design rationale, thermally activated systems have also been widely applied in antitumor therapy, including thermosensitive nanocomposite hydrogels for localized sustained chemotherapy (Zuo et al., 2024), temperature-responsive liposomal platforms for heat-triggered drug release and immune microenvironment modulation (Fu et al., 2023). In this study, the apparent “thermoresponsiveness” conferred by pDA did not originate from a classical thermally induced phase-transition behavior, but from NIR-triggered photothermal transduction. As a melanin-like polymer, pDA exhibits broad absorption from the ultraviolet to near-infrared region and efficiently converts absorbed light into localized heat through non-radiative energy dissipation (Zou et al., 2025). We found that pDA coating endowed EcN-Gadabc@pDA with stable NIR-responsive photothermal properties, enabling efficient conversion of external optical stimulation into local thermal cues. These thermal cues further activated the thermosensitive expression module, thereby enhancing GABA release and enabling precise spatiotemporal regulation of metabolic output. Previous studies have reported stronger photothermal performance for optimized pDA-based nanomaterials. For example, pDA nanotubes dispersed at 250 μg/mL exhibited a temperature increase of approximately 30 °C under 808 nm NIR-laser irradiation at 1 W/cm^2^ (Sun and Davis, 2019), which was higher than that observed for EcN-Gadabc@pDA in this study. This difference is likely related to variations in chemical composition, polymerization degree, aggregation state, particle size and composite structure, all of which are known to affect the photothermal behavior of melanin-like materials (Zou et al., 2025). Nevertheless, the photothermal effect achieved in our system was sufficient to activate the thermosensitive circuit and support controllable GABA production.

Targeted delivery and protective coatings are critical for oral probiotic therapy. Our results showed that the pDA layer formed a uniform nanoscale shell that significantly improved bacterial survival under simulated gastric, intestinal and bile salt conditions, while prolonging intestinal retention through enhanced mucosal adhesion. Moreover, the pDA surface provided a versatile platform for further functionalization. Consistent with our findings, a co-deposited polydopamine (pDA)/poly-L-lysine coating significantly increased bacterial survival in simulated gastrointestinal fluids, enhanced mucosal adhesion and improved therapeutic outcomes *in vivo* (Zhu et al., 2024). Given the feasibility of antibody-based conjugation strategies (Dixit et al., 2025), we further conjugated a pepT1 targeting peptide to the coating to promote small-intestinal targeting. *In vivo* imaging confirmed prolonged gastrointestinal retention of EcN-Gadabc@pDA-pepT1. PepT1 is a proton-dependent oligopeptide transporter highly expressed on the brush-border membrane of small-intestinal epithelial cells, where it mediates the high-capacity transport of dipeptides and tripeptides, driven by the transmembrane proton gradient. PepT1 can recognize and transport structurally diverse oral compounds, making it an attractive target for oral delivery (Killer et al., 2021). The conjugation between the pDA coating and the pepT1 targeting peptide is mainly attributed to covalent coupling and non-covalent adsorption, particularly involving reactions between quinone groups on the pDA surface and amino/thiol groups on the targeting peptide (Alfieri et al., 2023). Consistent with our strategy, a previous study achieved small-intestinal targeted delivery of engineered bacteria by combining a pepT1 targeting peptide with chitosan–sodium alginate hydrogel microspheres (Yang et al., 2020). These findings indicate that pDA-based nanocoating is an effective strategy for protecting engineered probiotics and improving their oral delivery and intestinal colonization.

Gut-derived GABA may influence brain function through multiple peripheral mechanisms, including both neural and immunomodulatory pathways. Vagal afferents are one of the key conduits for gut-to-brain communication, relaying intestinal signals to the nucleus tractus solitarius (NTS) and subsequently to emotion-related regions such as the locus coeruleus and amygdala (Décarie-Spain et al., 2024). Consistent with this anatomical framework, our study showed that EcN Gadabc@pDA-PepT1 plus NIR irradiation significantly improved anxiety-like behaviors in the repeated restraint stress model and was accompanied by reduced c-fos expression in the NTS and locus coeruleus. In addition to these brainstem changes, amygdala activity was also markedly reduced. The amygdala is a central hub for emotional processing. Hyperactive amygdala neurons have been associated with chronic stress-induced anxiety and depression and suppression of which directly produces anxiolytic effects (Babaev et al., 2018). Our results showed that EcN Gadabc@pDA-PepT1 plus NIR irradiation decreased c-fos expression in the basolateral, central and medial amygdala, indicating broad suppression of amygdala activation. To further corroborate this finding at the protein level, we performed ARC immunofluorescence staining—a marker of neuronal activation, and observed a consistent reduction in ARC-positive neurons in the basolateral, central and medial amygdala. The convergence of these two independent markers (c-fos and ARC) provides robust evidence that our engineered probiotic system suppresses amygdala reactivity.

Beyond neural pathways, our results showed that EcN-Gadabc@pDA-PepT1 plus NIR irradiation significantly decreased serum IL-1β, IL-6 and TNF-α levels, while increasing the anti-inflammatory cytokine IL-10 in comparison to the control groups receiving saline and EcN-GFP plus NIR, suggesting that release of GABA in the gut could produce a general anti-inflammatory effect. This is reasonable since GABA is increasingly recognized as an immune-regulatory molecule in the GI tract. Functional GABA receptors are ubiquitously expressed on multiple immune cell types and GABA signaling can suppress macrophage and T-cell activation, thereby reducing the production of pro-inflammatory cytokines such as IL-1β, IL-6 and TNF-α (Bhat et al., 2010; Prud’homme et al., 2015). In the intestinal microenvironment, GABA has also been implicated in maintaining mucosal immune homeostasis and limiting excessive inflammation (Sahoo et al., 2025). Elevated circulating pro-inflammatory cytokines can promote neuroinflammatory signaling and alter the activity of emotion-related brain regions, including the amygdala (Dantzer et al., 2008; Socała et al., 2021; Zheng et al., 2021). Notably, IL-10 has been reported to directly modulate GABAergic transmission in the central amygdala, thereby reversing anxiety-like behaviors (Patel et al., 2021). Although we did not directly assess IL-10 signaling in the amygdala, the increased serum IL-10 level in the treatment group, together with reduced amygdala c-fos and ARC activity, supports the possibility that peripheral anti-inflammatory responses contributed to the suppression of anxiety-related neural activation. These findings suggest that bacteria-derived GABA may exert both local and systemic anti-inflammatory effects, providing an additional mechanism that may contribute to the therapeutic benefit of this system.

In addition, the remodeling of the gut microbial ecosystem represents another potential mechanism underlying the therapeutic effect observed in this study. Gut microbiota dysbiosis has been closely linked to anxiety-related behaviors and microbial metabolites are increasingly recognized as important mediators of gut–brain communication (Foster and McVey Neufeld, 2013). In our study, 16S rRNA sequencing showed that treatment with the engineered strain partially restored gut microbial α-diversity and altered genus-level microbial composition. Notably, the relative abundance of *Clostridium* was significantly reduced. Because expansion of *Clostridium* has been associated with intestinal inflammation and anxiety- or depression-like behaviors (Jiang et al., 2015), this change suggests that EcN Gadabc@pDA-pepT1 may improve the intestinal microenvironment by suppressing potentially deleterious taxa and promoting microbial homeostasis. To further explore the functional potential of the remodeled microbiota, we performed PICRUSt2 functional prediction analysis. The EcN-GABA treatment group exhibited significant alterations in multiple COG categories, with notable upregulation of alpha-mannosidase and polygalacturonase. Alpha-mannosidase participates in the degradation of mannose-containing glycans, releasing simple sugars that can be fermented by gut bacteria into short-chain fatty acids (SCFAs), including butyrate (Boushra et al., 2025). Polygalacturonase degrades pectin to generate galacturonic acid, which is further metabolized by commensal bacteria to produce SCFAs, particularly butyrate and acetate (Xu et al., 2025). Butyrate has been well documented to exert anxiolytic effects via the gut–brain axis. (Boushra et al., 2024). These functional predictions provide directional evidence that the gut microbiota remodeling induced by EcN-GABA treatment contributes to the observed anxiolytic effect through enhanced SCFA-producing potential. This microbiota-regulating effect is also consistent with the intrinsic probiotic properties of the EcN chassis. EcN is known to support intestinal barrier integrity, regulate mucosal immunity and antagonize enteric pathogens, all of which are relevant to gut–brain axis homeostasis (Zyrek et al., 2007; Zhao et al., 2022). In line with this, our transcriptomic analysis further suggested that EcN colonization and GABA release contribute through distinct but complementary pathways. GABA-associated responses were enriched in pathways related to neuronal excitability, ion channel regulation and calcium signaling. Together, these findings support a synergistic model in which the EcN chassis establishes a favorable intestinal context, while stimulus-triggered GABA release provides a direct effector signal for gut–brain modulation, thereby jointly contributing to the anxiolytic-like effect of the engineered strain.

In summary, the photothermally responsive engineered probiotic EcN-Gadabc@pDA-PepT1 developed in this study enables precise, NIR-triggered release of GABA. Our data suggest that this system alleviates anxiety-like behavior in mice through coordinated regulation of gut–brain signaling, peripheral inflammatory responses and gut microbiota composition, leading to reduce amygdala activity. This strategy provides a proof-of-concept for precision intervention of gut–brain axis-mediated mood regulation and highlights the potential of light-controllable probiotics as a bioengineered platform for psychiatric disorders. Further refinement of the light-controllable system and validation in additional preclinical models may facilitate the development of safer and more effective non-invasive strategies for targeted modulation of mood disorders.

## Data Availability

The original contributions presented in the study are included in the article/Supplementary Material, further inquiries can be directed to the corresponding authors.
