# Supplementary material for: A light-responsive engineered probiotic system to alleviate anxiety via gut-brain axis
Source: Front Bioeng Biotechnol. 2026 Oct 8;14:1864003. doi: 10.3389/fbioe.2026.1864003 (PMC13687339; doi:10.3389/fbioe.2026.1864003)
Supplement: Supplementary file 1 [file Supplementaryfile1.docx]

Supplementary Material

# 1 Supplementary Figures and Tables

1.1 **Supplementary Figures**

**Supplementary Figure 1.** Temperature rise of EcN@pDA in SIF under NIR (60 min) and return to baseline after off.

**Supplementary Figure 2.** The transcript levels of GadA, GadB, and GadC in EcN-Gadabc at different temperatures were measured by qPCR.

**Supplementary Figure 3.** Serum levels in groups with or without NIR irradiation. All data are presented as mean ± SEM. *P < 0.05, **P < 0.01, ***P < 0.001, ****P < 0.0001.

**Supplementary Figure 4.** Assembly of pDA onto EcN. **(A)** Colonies of EcN and EcN@pDA. **(B)** Growth curves of EcN and EcN@pDA.

**Supplementary Figure 5.** The attachment of Alexa Fluor®647 pepT1 and EcN‑GFP@pDA to the small intestinal villi.

 **Supplementary Figure 6.** Levels of alanine aminotransferase (ALT), albumin (ALB), blood urea nitrogen (BUN), Creatinine (CREA), and Creatine Kinase (CK). All data are presented as mean ± SEM. *P < 0.05, **P < 0.01, ***P < 0.001, ****P < 0.0001.

**Supplementary Figure 7.**  PICRUSt2 functional prediction analysis. All data are presented as mean ± SEM. *P < 0.05, **P < 0.01, ***P < 0.001, ****P < 0.0001.

**Supplementary Figure 8.** Changes in c‑Fos‑positive cell counts in anxiety‑related brain regions following EcN‑Gadabc@pDA‑PepT1/NIR treatment. The regions examined include the infralimbic cortex (IL), claustrum (Cl), bed nucleus of the stria terminalis (BST), prelimbic cortex (PrL), lateral habenula (LHb), ventral hippocampal commissure (vhc), ventral tegmental area (VTA), locus coeruleus (LC), lateral hypothalamus (LH), and nucleus tractus solitarii (Sol). All data are presented as mean ± SEM. *P < 0.05, **P < 0.01, ***P < 0.001, ****P < 0.0001.

**Supplementary Figure 9.** Changes in Arc‑positive cell counts in anxiety‑related brain regions following EcN‑Gadabc@pDA‑PepT1/NIR treatment. The regions examined include the basolateral amygdala (BLA), central amygdala (CeA) and medial amygdala (MeA). All data are presented as mean ± SEM. *P < 0.05, **P < 0.01, ***P < 0.001, ****P < 0.0001.

1.2 **Supplementary Tables**

**Table 1.** Exact P-values, F-statistics or and post-hoc pairwise results.

**Tables 2.** Cohen’s d effect sizes for all primary behavioral endpoints.
